# Clinicopathological and Prognostic Value of Ki-67 Expression in Bladder Cancer: A Systematic Review and Meta-Analysis

**DOI:** 10.1371/journal.pone.0158891

**Published:** 2016-07-13

**Authors:** Yuejun Tian, Zhiming Ma, Zhaohui Chen, Mingguo Li, Zhiping Wu, Mei Hong, Hanzhang Wang, Robert Svatek, Ronald Rodriguez, Zhiping Wang

**Affiliations:** 1 Institute of Urology, Lanzhou University Second Hospital, Key Laboratory of Gansu Province for Urological Diseases, Clinical Center of Gansu Province for Nephro-urology, Lanzhou University, Lanzhou, China; 2 Department Gastroenterology, The Second Hospital of Lanzhou University, Lanzhou University, Lanzhou, China; 3 Department of Urology, University of Texas Health Science Center San Antonio, 7703 Floyd Curl Drive, San Antonio, Texas, United States of America; Catalan Institute of Oncology, SPAIN

## Abstract

**Background:**

Ki-67 is an established marker of cell proliferation, and the Ki-67 index correlates with the clinical course of several cancer types, including bladder cancer (BC). However, the clinicopathological and prognostic significance of Ki-67 in bladder cancer remains unclear. Therefore, we performed a systematic review and meta-analysis to clarify this relationship.

**Methods:**

A comprehensive literature search for relevant studies published up to February 1, 2016, was performed using PubMed, Cochrane Library, Embase and ISI Web of Knowledge. The effects of Ki-67 expression on survival outcome in patients with BC and BC subtypes were evaluated. Furthermore, the relationship between Ki-67 expression and the clinicopathological features of BC were assessed.

**Results:**

Thirty-one studies with 5147 bladder cancer patients were selected for evaluation. Ki-67 expression was significantly associated with shorter recurrence-free (HR 1.69, 95% CI: 1.33–2.14), progression-free (HR 1.89, 95% CI: 1.43–2.51), overall (HR 2.03, 95% CI: 1.31–3.16), and cancer-specific (HR 1.69, 95% CI: 1.47–1.95) survival. Moreover, whereas high expression was more common in high tumor stage, recurrence status, tumor size, there was no correlation between high Ki-67 expression and age, gender, smoking habits, and tumor number. Importantly, analysis of the different subgroups of BC suggested that significant correlations between high Ki-67 expression and survival outcome (recurrence-free/progression-free/overall/cancer-specific survival) are present only in European-American patients.

**Conclusion:**

The present results indicate that over-expression of Ki-67 is distinctly correlated with poor patient survival. Ki-67 may serve as a valuable biomarker for prognosis in BC patients, particularly in non-Asian BC patients. The results suggest no significant association between Ki-67 expression and BC prognosis in Asian patients. Further efforts are needed to fully clarify this relationship.

## Introduction

Bladder cancer (BC) is a common cancer of the urinary tract, with an estimated 429,800 new cases of BC and 165,100 deaths annually worldwide [1]. Clinically, bladder tumors are classified as non-muscle invasive bladder cancer (NMIBC)(Ta/T1) and muscle-invasive bladder cancer (MIBC)(T2-T4), NMIBC is also called superficial bladder cancer. Although many factors have been identified as risk factors for BC, such as smoking, age, obesity and diabetes, the pathogenesis of BC remains unclear [2, 3]. Cystoscopy and biopsy are the gold standards for the initial diagnosis of BC but are invasive, uncomfortable, and expensive [4, 5]. Therefore, novel biomarkers for early diagnosis, prognostic evaluation and effective treatment of BC are needed.

Ki-67 is a DNA-binding nuclear protein that is expressed throughout the cell cycle in proliferating but not quiescent (G0) cells [6]. Ki-67 is a predictive factor for tumor development, and its expression has been correlated with poor prognosis in several types of cancer [7–10]. However, the role of Ki-67 in the prognosis of BC remains controversial. Chen et al. [11] confirmed that Ki-67 was an independent predictor of tumor recurrence and progression in a study of 72 cases of NMIBC. Makboul and Gontero et al. [12, 13] demonstrated that Ki-67 was only an independent predictor of progression and not recurrence in NMIBC patients. Tanabe et al. [14] demonstrated that high Ki-67 expression status might facilitate the selection of chemoradiotherapy-based multimodal approaches in terms of prognosis and quality of life as a result of bladder preservation in MIBC patients. Studies have revealed that Ki-67 is not correlated with or an independent predictor of BC recurrence, progression, and death. For example, Acikalin et al. [15] reported that there was no correlation between Ki-67 and tumor recurrence, progression or tumor-related mortality in a study of 68 patients with stage T1 who underwent transurethral resection of the tumor.

The optimal approach to the interpretation and assessment of Ki-67 in clinical practice remains controversial among pathologists. In addition, the roles of Ki-67 expression and clinical significance in BC have not been thoroughly investigated. In this study, we performed a meta-analysis to explore the relationship between Ki-67 expression and its prognostic value in BC. This systematic review and meta-analysis was reported and performed in accordance with PRISMA guidelines (S3 Table) [16].

## Materials and Methods

### Study strategy

The PubMed, Cochrane, Embase and Web of Knowledge databases were searched systematically for relevant articles published up to February 1, 2016. Because the data in this study were extracted from previous studies, ethical approval from ethics committees was not required.

The search terms were ‘‘bladder,” ‘‘urothelial,” ‘‘cancer or tumor or neoplasm or carcinoma,” ‘‘expression,” ‘‘Ki-67 or Ki67 or MIB-1 or MIB 1”, and ‘‘prognosis or prognostic or outcome.” The criteria for eligibility were as follows: (1) Ki-67 expression evaluated in primary BC tissues; (2) evaluation of the relationship between Ki-67 expression and BC clinicopathological parameters and prognosis; and (3) sufficient information to estimate the hazard ratio (HR) of recurrence-free survival (RFS), progression-free survival (PFS), overall survival (OS), and cancer-specific survival (CSS) and a 95% confidence intervals (CIs). Papers containing any of the following were excluded: (1) duplicate literature or duplicate data presented at conferences; (2) reviews, no available data, or abstract only; (3) studies of cancer cell lines and animal models; and (4) insufficient data to obtain HR and its standard error. For overlapping articles, only the highest-quality and most-recent literature were retained.

### Data extraction and methodological assessment

The following information was recorded for each study: the first author’s name, publication year, sample source, number of cases, median or mean of patient age, gender, cancer stage, antibody source and dilution, percentage rate of expression, and follow-up period. We preferred to collect multivariate analysis data. If data were not available, data from univariate analyses of survival outcomes were extracted. All data were extracted by two independent observers (ZMM and ZHC). The quality of the selected articles was assessed according to the Newcastle-Ottawa Scale (NOS) criteria [17]. If data could not be obtained from the literature, we regarded the related data as not available.

### Statistical analysis

The statistical analysis was conducted using Review Manager 5.3 (Cochrane Collaboration, Oxford, UK) and STATA 14.0 (Stata Corporation, TX). HRs and 95% CIs were used to evaluate the relationships between Ki-67 expression and RFS, PFS, OS, and CSS rates. ORs (odds ratios) and 95% CIs were used to estimate the relationships between Ki-67 expression and clinicopathological parameters, including age, sex status, tumor stage, recurrence status, tumor number, and tumor size. The statistical significance of the pooled ORs and HRs was evaluated by the Z test. Heterogeneity among the studies was evaluated with Cochran’s Q test and I^2^ tests [18]. When the I^2^ statistic results were 0–50%, a fixed-effect model was used to calculate parameters. If the I^2^ statistic results were 50–100%, a random-effects model was considered more appropriate than a fixed-effects model. A p value < 0.05 was considered statistically significant. Funnel plots and Begg’s test were used to evaluate potential publication bias [19].

## Results

### Study characteristics

**Fig 1:**
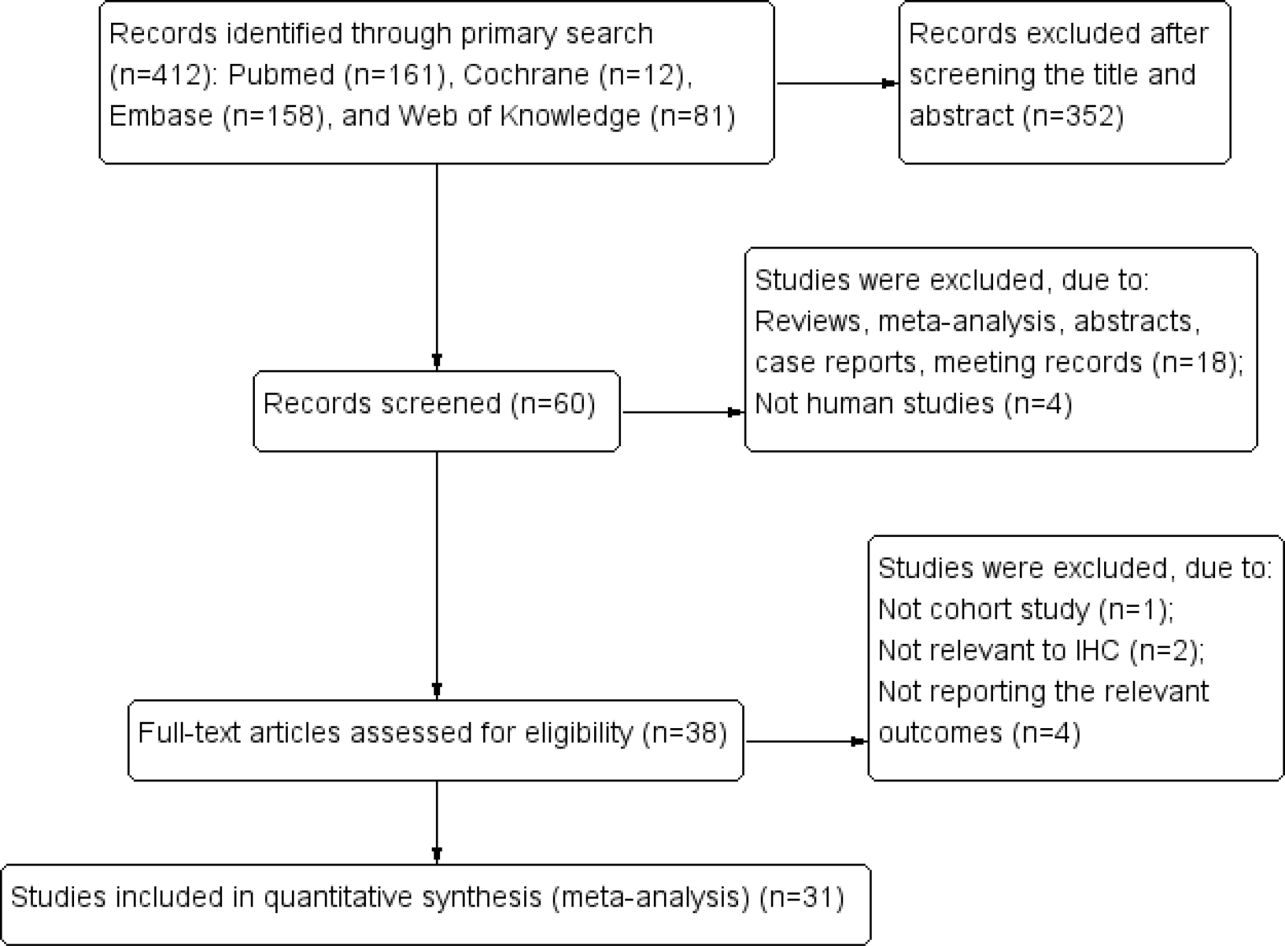
Flow chart shows study selection procedure.

Our search strategy initially identified 412 articles. Following deduplication (n = 60), the two reviewers independently screened the identified titles and abstracts. After manually screening the titles and abstracts, 22 studies were excluded because they were case reports (n = 2), review articles (n = 6), conference abstracts (n = 4), meta-analysis (n = 2) or studies irrelevant to the human studies (n = 8). Seven articles were ultimately excluded due to overlap with previously reported studies (n = 4). Thus, 31 articles published from 2001 to 2016 were included in the final meta-analysis [20–50] (Fig 1).

The main characteristics of the 31 studies included in our meta-analysis are presented in S1 Table. Of the 31 studies, 5 were conducted in America, five in Germany, five in China, three in Greece, three in Spain, three in Korea, two in Italy, two in Japan, and one each in Portugal, Switzerland, and the UK. In 5 of the 31 studies, patients received intravesical BCG therapy. The follow-up period of the studies ranged from 2 months to 124 months. The age of the patients ranged from 21 to 97 years, and the overall proportion of males was 80.33%.

Positive/high Ki-67 expression was defined by immunohistochemistry (IHC) using different antibodies and cut-off values (range, 5–55%) (S2 Table).

Of the 31 studies, 23 provided HRs and 95% CI values directly. Six papers provided the relative risk (RR), and two articles provided OR values, which we used to estimate HR. Of the 31 studies, a significant association between high Ki-67 expression and poor RFS, PFS, OS and CSS was demonstrated in five [22, 26, 34, 35, 48], five [31, 36, 43, 46, 48], six [20, 24, 28, 29, 31, 42] and seven studies [27, 30, 33, 34, 42, 46, 47], respectively. Of the literature, eleven, five, three and two studies linking Ki-67 expression with poor RFS [21, 25, 37–41, 43–45], PFS[21, 25, 41, 44, 49], OS [23, 32, 41] and CSS [43, 50], respectively, lacked statistical significance.

### Correlation of high Ki-67 expression with RFS in bladder cancer

**Fig 2:**
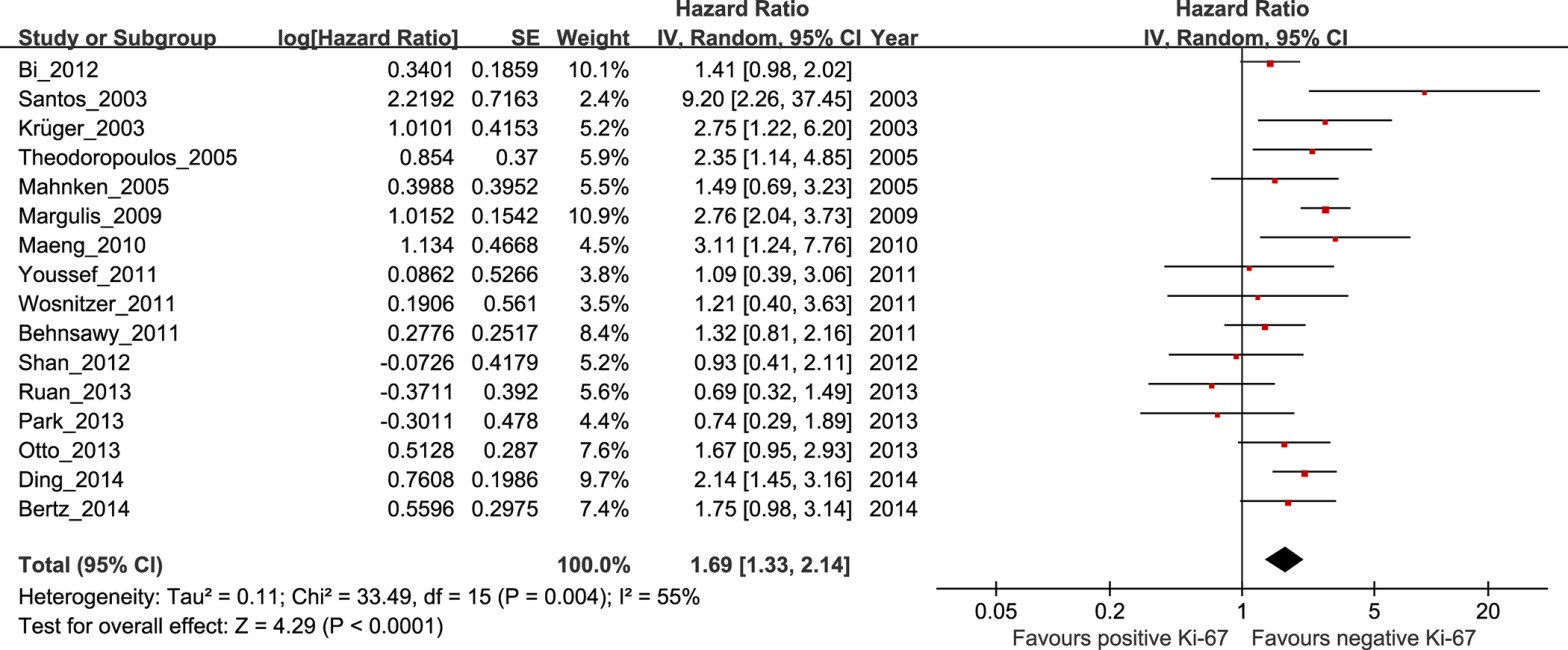
The hazard ratio (HR) of Ki-67 expression associated with RFS in all bladder cancer patients.

Of the 16 studies investigating the association between Ki-67 expression and RFS, 7 involved Asian patients (n = 2163), and 9 involved non-Asian patients (n = 610). The overall HR for BC patients was 1.69 (95% CI 1.33–2.14, P < 0.0001, n = 2773), with significant heterogeneity (I^2^ = 55%, P = 0.004; Fig 2 and Table 1). Subgroup analyses indicated that the risk was significant in non-Asian patients (HR 2.23, 95% CI 1.82–2.73, P < 0.00001) with heterogeneity (I^2^ = 33%, P = 0.16), but not in Asian patients (HR 1.36, 95% CI 0.97–1.90, P = 0.07), with significant heterogeneity (I^2^ = 55%, P = 0.04).

**Table 1:** Results of subgroup analysis of the association between Ki-67 expression and RFS/PFS/OS/CSS of bladder cancer.

	Outcome	Studies (n)	Patients	HR	95%CI	P value	Model	Heterogeneity
								Chi^2^, I^2^, P value
**RFS**	**All study**	16	2773	1.69	1.33–2.14	0.000	Random	33.49, 55%, 0.004
	**Asian**	7	2163	1.36	0.97–1.90	0.07	Random	13.37, 55%, 0.04
	**Non-Asian**	9	610	2.23	1.82–2.73	0.000	Fixed	11.85, 33%, 0.16
	**Stage T1**	6	927	1.45	1.09–1.93	0.01	Fixed	8.58, 42%, 0.13
	**Stage Ta-T1**	6	774	1.99	1.54–2.57	0.000	Fixed	9.26, 46%, 0.10
	**Stage Ta/1-T4**	4	1072	1.56	0.91–2.66	0.11	Random	12.52, 76%, 0.006
	**UBC**	14	2560	1.79	1.40–2.28	0.000	Random	28.88, 55%, 0.007
	**SCC**	1	152	1.09	0.39–3.08	0.87	Fixed	-
	**BCG**	5	522	1.63	1.20–2.23	0.002	Fixed	4.42, 10%, 0.35
**PFS**	**All study**	10	1694	1.89	1.43–2.51	0.000	Fixed	16.34, 45%, 0.06
	**Asian**	4	618	1.35	0.48–3.82	0.57	Random	11.41, 74%, 0.01
	**Non-Asian**	6	1076	2.05	1.45–2.89	0.000	Fixed	4.33, 0%, 0.50
	**Stage T1**	5	799	1.78	1.22–2.60	0.003	Fixed	6.42, 38%, 0.17
	**Stage Ta-T1**	4	799	2.80	1.75–4.49	0.000	Fixed	1.04, 0%, 0.79
	**Stage Ta-T4**	1	96	0.57	0.22–1.49	0.253	Fixed	-
**OS**	**All study**	9	1159	2.03	1.31–3.16	0.002	Random	40.37, 80%, 0.000
	**Asian**	2	241	2.97	0.19–47.15	0.44	Random	12.21, 92%, 0.0005
	**Non-Asian**	7	918	1.96	1.26–3.06	0.003	Random	27.12, 78%, 0.0001
	**Stage Ta-T1**	4	638	2.76	1.81–4.20	0.001	Fixed	1.05, 0%, 0.79
	**Stage T2-T4**	1	82	2.33	0.99–5.43	0.05	Fixed	-
	**Stage Ta/1-T4**	4	439	1.40	0.82–2.40	0.22	Random	15.33, 80%, 0.002
**CSS**	**All study**	9	2528	1.69	1.47–1.95	0.000	Fixed	10.42, 23%, 0.24
	**Asian**	1	103	1.58	0.56–4.47	0.38	Fixed	-
	**Non-Asian**	8	2425	1.69	1.47–1.95	0.000	Fixed	10.41, 33%, 0.17
	**Stage T1**	3	695	2.86	1.16–7.02	0.02	Random	4.95, 60%, 0.08
	**Stage Ta-T1**	1	192	3.46	1.22–9.80	0.01	Fixed	-
	**Stage T2-T4**	1	73	4.70	1.14–19.28	0.032	Fixed	-
	**Stage Ta/1-T4**	5	1641	1.61	1.39–1.87	0.000	Fixed	0.73, 0%, 0.95

BCG: bacillus Calmette-Guerin; CSS: cancer-specific survival; Fixed: Fixed, Inverse Variance model; HR: hazard ratio; I^2^: I-squared; OS: overall survival; PFS: progression-free survival; Random: Random, I-V heterogeneity model; RFS: recurrence-free survival; SCC: squamous cell carcinoma; UBC: urothelial bladder cancer.

Next, subgroups including tumor stage (six studies for stage T1, six for stages Ta-T1, and four for stages Ta/1-T4) and type of BC (14 studies for urothelial bladder cancer and 1 for squamous cell carcinoma) were analyzed. The analyses indicated that high Ki-67 expression was associated with shorter RFS in stage T1 and stages Ta-T1 patients (HR 1.45, 95% CI 1.09–1.93, P = 0.01; and HR 1.99, 95% CI 1.54–2.57, P < 0.00001, respectively) with heterogeneity (I^2^ = 42%, P = 0.13; and I^2^ = 46%, P = 0.10, respectively), but no association with shorter RFS was observed in patients in stages Ta/1-T4 (HR 1.56, 95% CI 0.91–2.66, P = 0.11). Moreover, our analyses revealed that Ki-67 expression was associated with shorter RFS in urothelial bladder cancer (HR 1.79, 95% CI 1.40–2.28, P < 0.00001). No significant association was observed between Ki-67 expression and squamous cell carcinoma (HR 1.09, 95% CI 0.39–3.08, P = 0.87). Furthermore, Ki-67 expression was an independent prognostic factor for BC treated with BCG therapy (HR, 1.63; 95% CI, 1.20–2.23; P = 0.002) (Table 1).

### Relationships between Ki-67 expression and RFS in bladder cancer using different cut-off values

Subgroup analysis demonstrated that the relationship between Ki-67 expression and RFS was not significant using different Ki-67 cut-off values (10%, 25%, 50%). The pooled HRs and 95% CIs were as follows: 1.56 (95% CI 1.13–2.16) vs. 1.68 (95% CI 1.27–2.21) for a cut-off value of 10%, 1.61(95% CI 1.16–2.22) vs. 1.97 (95% CI 1.48–2.62) for a cut-off value of 25%, and 1.65 (95% CI 1.27–2.15) vs. 1.99 (95% CI 1.14–3.49) for a cut-off value of 50% (S1–S3 Figs and S2 Table).

### Correlation between high Ki-67 expression and PFS in bladder cancer

**Fig 3:**
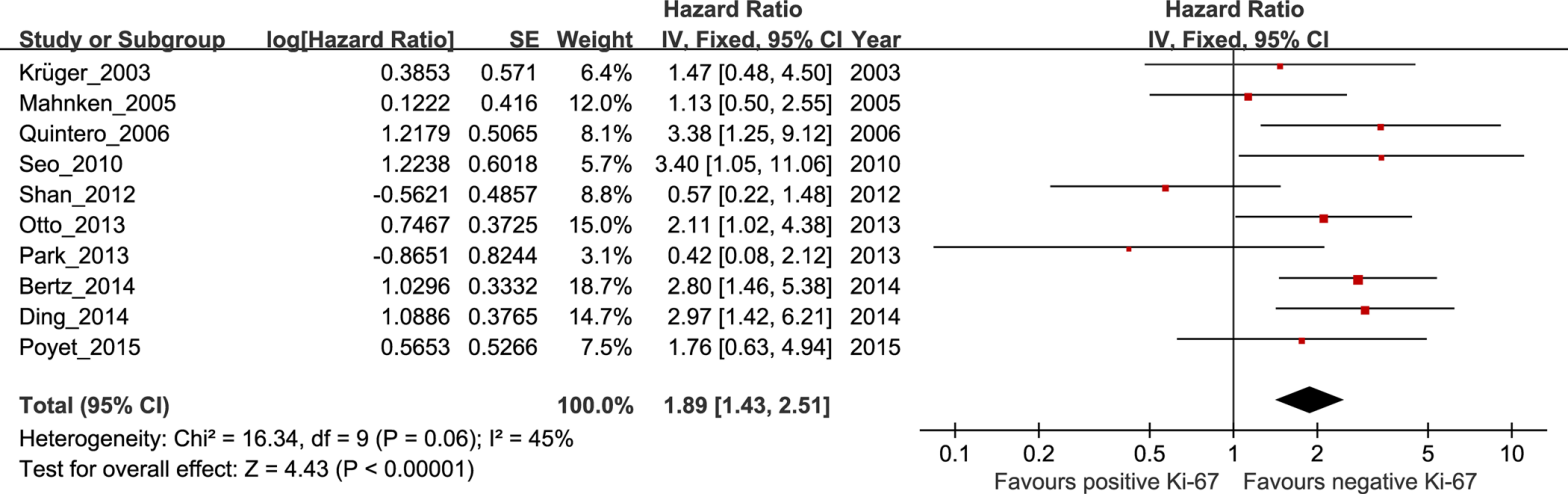
The hazard ratio (HR) of Ki-67 expression associated with PFS in all bladder cancer patients.

The pooled HR and 95% CI for RFS provided in ten studies was 1.89, 95% CI 1.43–2.51, P < 0.0001, with heterogeneity (I^2^ = 45%, P = 0.06; Fig 3 and Table 1). The risk was significant in non-Asian patients but not in Asian patients, and the combined HRs and 95% CIs were as follows: HR 2.05, 95% CI 1.45–2.89, P < 0.0001; and HR 1.35, 95% CI 0.48–3.82, P = 0.57, respectively. Further subgroup analysis indicated that the risk was higher in the very early stage (stages Ta-T1) compared with stage T1, with the following combined HRs and 95% CIs: HR 2.80, 95% CI 1.75–4.49, P < 0.00001; HR 1.78, 95% CI 1.22–2.60, P = 0.003, respectively. But no significant association with PFS was observed in patients in stages Ta-T4, and the combined HRs and 95% CIs were as follows: HR 0.57, 95% CI 0.22–1.49, P = 0.253.

### Correlation of high Ki-67 expression with OS and CSS in bladder cancer

The pooled HR for OS provided in nine studies indicated that Ki-67 expression was associated with worse survival in BC patients (HR = 2.03, 95% CI 1.31–3.16; P = 0.002), with heterogeneity (I^2^ = 80%, P < 0.0001; S4 Fig and Table 1). Subgroup analysis demonstrated that the risk was significant in non-Asian patients but not in Asian patients, and the combined HRs and 95% CIs were as follows: HR 1.96, 95% CI 1.26–3.06, P = 0.003; and HR 2.97, 95% CI 0.19–47.15, P = 0.44, respectively. Next, subgroups including tumor stage (four studies for stages Ta-T1, one for stages T2-T4, and four for stages Ta/1-T4) were analyzed. The analyses indicated that high Ki-67 expression was associated with shorter OS in stages Ta-T1 patients (HR 2.76, 95% CI 1.81–4.20, P = 0.001) with heterogeneity (I^2^ = 0%, P = 0.79), but no association with shorter OS was observed in patients in stages T2-T4 and stages Ta/1-T4 (HR 2.33, 95% CI 0.99–5.43, P = 0.05; and HR 1.40, 95% CI 0.82–2.40, P = 0.22, respectively).

Similarly, the pooled HR for CSS provided in nine studies indicated that Ki-67 expression was associated with worse survival in BC patients (HR = 1.69, 95% CI 1.47–1.95; P < 0.0001), with heterogeneity (I^2^ = 23%, P = 0.24; S5 Fig and Table 1). Subgroup analysis demonstrated the risk was significant in non-Asian patients but not in Asian patients, and the combined HRs and 95% CIs were as follows: HR 1.69, 95% CI 1.47–1.95, P < 0.0001; and HR 1.58, 95% CI 0.56–4.47, P = 0.38, respectively. Next, subgroups including tumor stage (three studies for stage T1, one for stages Ta-T1, one for stages T2-T4, and five for stages Ta/1-T4) were analyzed. The analyses indicated that high Ki-67 expression was associated with shorter OS in stage T1, stages Ta-T1, stages T2-T4, and stages Ta/1-T4 patients (HR 2.86, 95% CI 1.16–7.02, P = 0.02; HR 3.46, 95% CI 1.22–9.80, P = 0.01; HR 4.70, 95% CI 1.14–19.28, P = 0.032; and HR 1.61, 95% CI 1.39–1.87, P < 0.00001, respectively).

### Relationships between Ki-67 expression and clinicopathological parameters

In this meta-analysis, the relationships between clinicopathological characteristics such as age, gender, smoking habits, tumor stage, recurrence status, tumor number, and tumor size and elevated Ki-67 expression were compared on the basis of 31 studies. The results of the meta-analysis revealed significant associations between high Ki-67 expression and higher tumor stage (Ta vs. T1; Ta/1 vs. T2-4), recurrence status, and larger tumor size. The combined ORs and 95% CIs were as follows: OR 0.29, 95% CI 0.19–0.42, P < 0.00001; OR 0.30, 95% CI 0.09–1.02, P = 0.05; OR 0.43, 95% CI 0.20–0.90, P = 0.02; and OR 1.80, 95% CI 1.26–2.56, P = 0.001, respectively. However, significant associations between Ki-67 and age, gender, smoking habits, and tumor number were not observed in BC patients. The combined ORs and 95% CIs were as follows: OR 1.02, 95% CI 0.41–2.54, P = 0.97; OR 1.09, 95% CI 0.83–1.43, P = 0.55; OR 1.28, 95% CI 0.86–1.89, P = 0.22; and OR 1.28, 95% CI 0.60–2.77, P = 0.52, respectively (Table 2).

**Table 2:** Results of subgroup analysis of the association between Ki-67 expression and clinicopathological parameters.

Outcome of interest	Studies (n)	Patients	OR	95%CI	P value	Model	Heterogeneity
							Chi^2^, I^2^, P value
**Age (≥65 vs. <65)**	2	293	1.02	0.41–2.54	0.97	Random	2.03, 51%, 0.15
**Gender (Male vs. Female)**	6	1551	1.09	0.83–1.43	0.55	Fixed	3.59, 0%, 0.61
Asian	3	522	0.89	0.56–1.43	0.63	Fixed	1.75, 0%, 0.42
Non-Asian	3	1029	1.20	0.86–1.68	0.29	Fixed	0.87, 0%, 0.65
**Smoke habits (Smoke vs. Non-smoke)**	1	588	1.28	0.86–1.89	0.22	Fixed	-
**Ta vs. T1**	4	570	0.29	0.19–0.42	0.000	Fixed	3.20, 6%, 0.36
**Ta-1 vs. T2-4**	3	1010	0.30	0.09–1.02	0.05	Random	12.97, 85%, 0.002
**Recurrence vs. No recurrence**	6	897	0.43	0.20–0.90	0.02	Random	18.64, 73%, 0.002
**Multiple vs. Single**	3	522	1.28	0.60–2.77	0.52	Random	6.28, 68%, 0.04
**Tumor size (<3 vs. ≥3cm)**	4	686	1.80	1.26–2.56	0.001	Fixed	3.46, 13%, 0.33

Fixed: Fixed, Inverse Variance model; I^2^: I-squared; OR: odd ratio; Random: Random, I-V heterogeneity model.

### Publication bias

**Fig 4:**
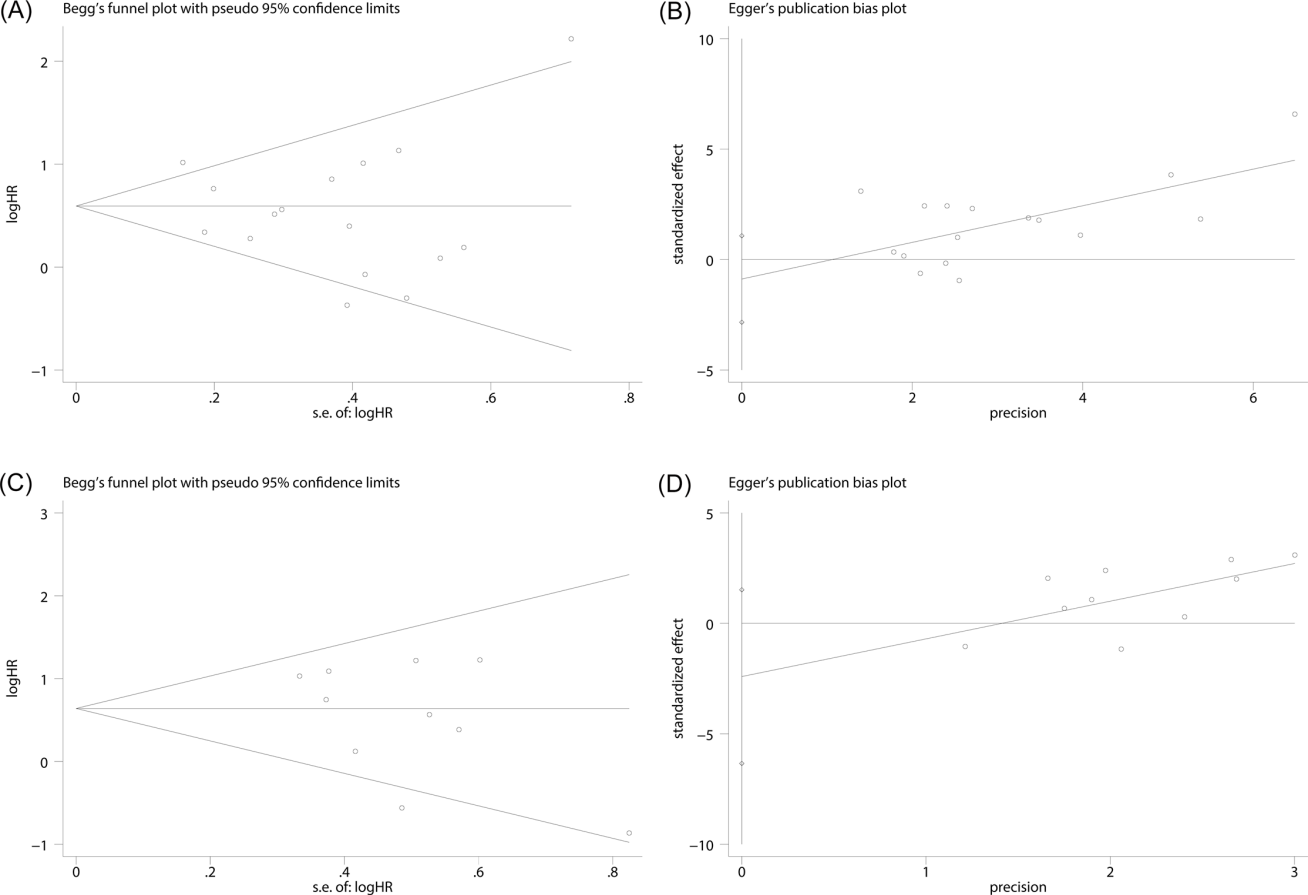
Funnel plots were used to evaluate publication bias on RFS and PFS. (A) Begg’s test was not significant intending no significant bias was observed on RFS. (B) Egger’s test was not significant intending no significant bias was observed on RFS. (C) It showed no publication bias on PFS in Begg’s test, (D) It showed no publication bias on PFS in Egger’s test.

Publication bias was conducted by Begg’s test for RFS and PFS of bladder carcinoma, with P values of 0.964 and 0.152, respectively. (Fig 4A and 4C). Quantitative assessment by Egger’s test for RFS and PFS suggested that our analyses were stable (P = 0.350, P = 0.195) (Fig 4B and 4D).

## Discussion

Increasing evidence indicates that BC genomes exhibiting the most complex alterations are associated with a high Ki-67 proliferation index [51]. Pichu et al. [52] reported that in BC cells, prior exposure to anti-Ki-67 siRNA induces tumor cells to undergo curcumin-induced growth arrest and apoptosis by non-p53 and non-p21-dependent signaling pathways, which may be useful for gene therapy. Wang et al. [53] reported that the combined effects of TP53 and Ki-67 revealed predictive value for NMIBC recurrence. However, the relationship between Ki-67 and outcome remains unclear, and the roles and clinical significance of Ki-67 expression in BC have not been thoroughly investigated [54].

In the present study, the analyses of the pooled data indicated that (1) BC patients with high Ki-67 expression had a lower survival rate; (2) high Ki-67 expression was associated with the more aggressive clinical stage and larger tumor size in BC patients; (3) aberrant Ki-67 expression was higher in recurrent BC than in non-recurrent BC; (4) Ki-67 expression was not strongly associated with age, gender, and tumor number in BC patients; (5) a strong relationship between poor prognostic indicators and Ki-67 expression was established only for European-American patients. The correlation between Ki-67 expression and survival outcome (RFS/PFS/OS/CSS) did not reach statistical significance in Asian patients. Our study provides insights on the results of individual studies focused on the hypothesis that Ki-67 is a prognostic factor for BC and suggests that adjuvant therapy may be helpful in the high-risk subgroup of patients. Although further validation and investigation are needed, these data provide new insights on the biological aggressiveness of BC in Asian versus in non-Asian patients.

The biological mechanism of Ki-67 explains its prognostic significance in BC. Ki-67 is an index of cell proliferation and a measure of cell growth fraction during the G1, S, G2 and M stages of the cell cycle and is widely applied in immunohistochemistry (IHC) to estimate the activities of cell proliferation in many cancers. Some researches investigated the relationships between the Ki-67 and distant metastases [55, 56]. They found that Ki-67 expression was upregulated in the transforming growth factor-β1 (TGF-β1) treated tumors, and TGF-β1 promotes EMT (epithelial-to-mesenchymal transition), migration, and invasion in bladder cancer cells [57]. Furthermore, it was showed that highly Ki-67 may induce EMT by increasing the expression of vimentin, which enhances cancer cell invasion and metastatic [58].

The present meta-analysis is the first study to systematically evaluate the associations between Ki-67 expression and clinicopathological features and prognostic factors in BC. Ki-67 can be considered an oncogene, and its activation may contribute to tumor progression and poor prognosis. Based on this meta-analysis, we suggest that Ki-67 expression in BC tends to indicate a poor prognosis.

Several limitations of this study must be acknowledged. In the included studies, the antibodies used to detect Ki-67 expression were not identical (anti-Ki67 mAb and anti-MIB-1 mAb). The definitions of the cut-off value also differed. Clinical factors such as race, age, and the use of different chemotherapies in each study may also be sources of bias. Non-English studies, unpublished studies, and studies that did not provide sufficient data to calculate HRs were not included in the assessment of the predictive value of Ki-67 for survival. These approaches may have produced errors due to the inclusion of inaccurate readings. Finally, although we included 31 studies comprising 5147 cases in this meta-analysis, few studies were categorized for subgroup analysis, and several survival subgroup analyses data lack. Therefore, more well-designed and large-scale trials are needed to confirm these findings.

In conclusion, our meta-analysis confirmed the significant associations between Ki-67 expression and clinicopathological features and prognostic factors in BC. Although subgroup analysis indicated no significant association between Ki-67 expression and BC prognosis in Asian patients. Our meta-analysis demonstrates that Ki-67 has a detrimental effect on clinicopathological features and recurrence status in BC. Therefore, Ki-67 could serve as an independent prognostic factor of RFS, PFS, OS and CSS in European-American patients. Ki-67 may be a novel candidate for BC genotyping and an indicator for predicting the prognosis of BC patients.

## Supporting Information

S1 Fig Cutoff value ≥ 10% and cutoff value < 10%. **HR of Ki-67 expression associated with RFS in all BC patients subgroup.** Abbreviations: HR, hazard ratio; RFS, recurrence-free survival; BC, bladder cancer.
(TIF)

S2 Fig Cutoff value ≥ 25% and cutoff value < 25% **. HR of Ki-67 expression associated with RFS in all BC patients subgroup.** Abbreviations: HR, hazard ratio; RFS, recurrence-free survival; BC, bladder cancer.
(TIF)

S3 Fig Cutoff value ≥ 50% and cutoff value < 50% **. HR of Ki-67 expression associated with RFS in all BC patients subgroup**. Abbreviations: HR, hazard ratio; RFS, recurrence-free survival; BC, bladder cancer.
(TIF)

S4 Fig HR of Ki-67 expression associated with OS in all BC patients. Abbreviations: HR, hazard ratio; OS, overall survival; BC, bladder cancer.
(TIF)

S5 Fig HR of Ki-67 expression associated with CSS in all BC patients. Abbreviations: HR, hazard ratio; CSS, cancer-specific survival; BC, bladder cancer.
(TIF)

S1 Table Summary of the characteristics of enrolled studies. (DOCX)

S2 Table HR values of RFS of BC subgroups depended on cutoff value. (DOCX)

S3 Table PRISMA 2009 checklist. (DOC)

S4 Table The raw data of Fig 4. (RAR)

## References

[1] Torre LA, Bray F, Siegel RL, Ferlay J, Lortet-Tieulent J, Jemal A. Global cancer statistics, 2012. CA Cancer J Clin. 2015; 65:87–108. 10.3322/caac.21262 25651787

[2] Freedman ND, Silverman DT, Hollenbeck AR, Schatzkin A, Abnet CC. Association between smoking and risk of bladder cancer among men and women. JAMA. 2011; 306:737–45. 10.1001/jama.2011.1142 21846855 PMC3441175

[3] Cancer Genome Atlas Research Network. Comprehensive molecular characterization of urothelial bladder carcinoma. Nature. 2014; 507:315–22. 10.1038/nature12965 24476821 PMC3962515

[4] Lotan Y, Svatek RS, Sagalowsky AI. Should we screen for bladder cancer in a high-risk population?: A cost per life-year saved analysis. Cancer. 2006; 107:982–90. 10.1002/cncr.22084 16862567

[5] Avritscher EB, Cooksley CD, Grossman HB, Sabichi AL, Hamblin L, Dinney CP, et al Clinical model of lifetime cost of treating bladder cancer and associated complications. Urology. 2006; 68:549–53. 10.1016/j.urology.2006.03.062 16979735

[6] Schluter C, Duchrow M, Wohlenberg C, Becker MH, Key G, Flad HD, et al The cell proliferation-associated antigen of antibody Ki-67: a very large, ubiquitous nuclear protein with numerous repeated elements, representing a new kind of cell cycle-maintaining proteins. J Cell Biol. 1993; 123:513–22. 8227122 10.1083/jcb.123.3.513 PMC2200129

[7] Krabbe LM, Bagrodia A, Haddad AQ, Kapur P, Khalil D, Hynan LS, et al Multi-institutional validation of the predictive value of Ki-67 in patients with high grade urothelial carcinoma of the upper urinary tract. J Urol. 2015; 193:1486–93. 10.1016/j.juro.2014.11.007 25451830

[8] Shui R, Yu B, Bi R, Yang F, Yang W. An interobserver reproducibility analysis of Ki67 visual assessment in breast cancer. PLoS One. 2015; 10:e0125131 10.1371/journal.pone.0125131 25932921 PMC4416820

[9] Wen S, Zhou W, Li CM, Hu J, Hu XM, Chen P, et al Ki-67 as a prognostic marker in early-stage non-small cell lung cancer in Asian patients: a meta-analysis of published studies involving 32 studies. BMC Cancer. 2015; 15:520 10.1186/s12885-015-1524-2 26174366 PMC4502553

[10] Gayed BA, Youssef RF, Bagrodia A, Darwish OM, Kapur P, Sagalowsky A, et al Ki67 is an independent predictor of oncological outcomes in patients with localized clear-cell renal cell carcinoma. BJU Int. 2014; 113:668–73. 10.1111/bju.12263 23937277

[11] Chen JX, Deng N, Chen X, Chen LW, Qiu SP, Li XF, et al A novel molecular grading model: combination of Ki67 and VEGF in predicting tumor recurrence and progression in non-invasive urothelial bladder cancer. Asian Pac J Cancer Prev. 2012; 13:2229–34. 22901199 10.7314/apjcp.2012.13.5.2229

[12] Makboul R, Refaiy AE, Badary FA, Abdelkawi IF, Merseburger AS, Mohammed RA. Expression of survivin in squamous cell carcinoma and transitional cell carcinoma of the urinary bladder: a comparative immunohistochemical study. Korean J Urol. 2015; 56:31–40. 10.4111/kju.2015.56.1.31 25598934 PMC4294853

[13] Gontero P, Gillo A, Fiorito C, Oderda M, Pacchioni D, Casetta G, et al Prognostic factors of 'high-grade' Ta bladder cancers according to the WHO 2004 classification: are these equivalent to 'high-risk' non-muscle-invasive bladder cancer? Urol Int. 2014; 92:136–42. 10.1159/000351961 24080613

[14] Tanabe K, Yoshida S, Koga F, Inoue M, Kobayashi S, Ishioka J, et al High Ki-67 Expression Predicts Favorable Survival in Muscle-Invasive Bladder Cancer Patients Treated With Chemoradiation-Based Bladder-Sparing Protocol. Clin Genitourin Cancer. 2015; 13:e243–51. 10.1016/j.clgc.2015.03.002 25936588

[15] Acikalin D, Oner U, Can C, Acikalin MF, Colak E. Predictive value of maspin and Ki-67 expression in transurethral resection specimens in patients with T1 bladder cancer. Tumori. 2012; 98:344–50. 10.1700/1125.12403 22825520

[16] Moher D, Liberati A, Tetzlaff J, Altman DG, Prisma Group. Preferred reporting items for systematic reviews and meta-analyses: the PRISMA statement. Ann Intern Med. 2009; 151:264–9, W64. 19622511 10.7326/0003-4819-151-4-200908180-00135

[17] Stang A. Critical evaluation of the Newcastle-Ottawa scale for the assessment of the quality of nonrandomized studies in meta-analyses. Eur J Epidemiol. 2010; 25:603–5. 10.1007/s10654-010-9491-z 20652370

[18] Zintzaras E, Ioannidis JP. HEGESMA: genome search meta-analysis and heterogeneity testing. Bioinformatics. 2005; 21:3672–3. 10.1093/bioinformatics/bti536 15955784

[19] Peters JL, Sutton AJ, Jones DR, Abrams KR, Rushton L. Comparison of two methods to detect publication bias in meta-analysis. JAMA. 2006; 295:676–80. 10.1001/jama.295.6.676 16467236

[20] Kamai T, Takagi K, Asami H, Ito Y, Oshima H, Yoshida KI. Decreasing of p27(Kip1)and cyclin E protein levels is associated with progression from superficial into invasive bladder cancer. Br J Cancer. 2001; 84:1242–51. 10.1054/bjoc.2000.1736 11336477 PMC2363875

[21] Kruger S, Thorns C, Stocker W, Muller-Kunert E, Bohle A, Feller AC. Prognostic value of MCM2 immunoreactivity in stage T1 transitional cell carcinoma of the bladder. Eur Urol. 2003; 43:138–45. 12565771 10.1016/s0302-2838(02)00580-8

[22] Santos LL, Amaro T, Pereira SA, Lameiras CR, Lopes P, Bento MJ, et al Expression of cell-cycle regulatory proteins and their prognostic value in superficial low-grade urothelial cell carcinoma of the bladder. Eur J Surg Oncol. 2003; 29:74–80. 12559081 10.1053/ejso.2002.1371

[23] Gakiopoulou-Givalou H, Nakopoulou L, Panayotopoulou EG, Zervas A, Mavrommatis J, Giannopoulos A. Non-endothelial KDR/flk-1 expression is associated with increased survival of patients with urothelial bladder carcinomas. Histopathology. 2003; 43:272–9. 12940780 10.1046/j.1365-2559.2003.01690.x

[24] Lopez-Beltran A, Luque RJ, Alvarez-Kindelan J, Quintero A, Merlo F, Requena MJ, et al Prognostic factors in survival of patients with stage Ta and T1 bladder urothelial tumors: the role of G1-S modulators (p53, p21Waf1, p27Kip1, cyclin D1, and cyclin D3), proliferation index, and clinicopathologic parameters. Am J Clin Pathol. 2004; 122:444–52. 10.1309/LTFU-3UUM-BY09-5HUM 15362377

[25] Mahnken A, Kausch I, Feller AC, Kruger S. E-cadherin immunoreactivity correlates with recurrence and progression of minimally invasive transitional cell carcinomas of the urinary bladder. Oncol Rep. 2005; 14:1065–70. 16142373

[26] Theodoropoulos VE, Lazaris AC, Kastriotis I, Spiliadi C, Theodoropoulos GE, Tsoukala V, et al Evaluation of hypoxia-inducible factor 1alpha overexpression as a predictor of tumour recurrence and progression in superficial urothelial bladder carcinoma. BJU Int. 2005; 95:425–31. 10.1111/j.1464-410X.2005.05314.x 15679808

[27] Weiss C, Rodel F, Wolf I, Papadopoulos T, Engehausen DG, Schrott KM, et al Combined-modality treatment and organ preservation in bladder cancer. Do molecular markers predict outcome? Strahlenther Onkol. 2005; 181:213–22. 10.1007/s00066-005-1417-4 15827690

[28] Mylona E, Magkou C, Gorantonakis G, Giannopoulou I, Nomikos A, Zarogiannos A, et al Evaluation of the vascular endothelial growth factor (VEGF)-C role in urothelial carcinomas of the bladder. Anticancer Res. 2006; 26:3567–71. 17094484

[29] Galmozzi F, Rubagotti A, Romagnoli A, Carmignani G, Perdelli L, Gatteschi B, et al Prognostic value of cell cycle regulatory proteins in muscle-infiltrating bladder cancer. J Cancer Res Clin Oncol. 2006; 132:757–64. 10.1007/s00432-006-0123-7 16804724 PMC12161030

[30] Hilmy M, Campbell R, Bartlett JM, McNicol AM, Underwood MA, McMillan DC. The relationship between the systemic inflammatory response, tumour proliferative activity, T-lymphocytic infiltration and COX-2 expression and survival in patients with transitional cell carcinoma of the urinary bladder. Br J Cancer. 2006; 95:1234–8. 10.1038/sj.bjc.6603415 17024120 PMC2360556

[31] Quintero A, Alvarez-Kindelan J, Luque RJ, Gonzalez-Campora R, Requena MJ, Montironi R, et al Ki-67 MIB1 labelling index and the prognosis of primary TaT1 urothelial cell carcinoma of the bladder. J Clin Pathol. 2006; 59:83–8. 10.1136/jcp.2004.022939 16394286 PMC1860249

[32] Yurakh AO, Ramos D, Calabuig-Farinas S, Lopez-Guerrero JA, Rubio J, Solsona E, et al Molecular and immunohistochemical analysis of the prognostic value of cell-cycle regulators in urothelial neoplasms of the bladder. Eur Urol. 2006; 50:506–15; discussion 15. 10.1016/j.eururo.2006.03.027 16624482

[33] Shariat SF, Bolenz C, Godoy G, Fradet Y, Ashfaq R, Karakiewicz PI, et al Predictive value of combined immunohistochemical markers in patients with pT1 urothelial carcinoma at radical cystectomy. J Urol. 2009; 182:78–84; discussion 10.1016/j.juro.2009.02.125 19447418

[34] Margulis V, Lotan Y, Karakiewicz PI, Fradet Y, Ashfaq R, Capitanio U, et al Multi-institutional validation of the predictive value of Ki-67 labeling index in patients with urinary bladder cancer. J Natl Cancer Inst. 2009; 101:114–9. 10.1093/jnci/djn451 19141773

[35] Maeng YH, Eun SY, Huh JS. Expression of fibroblast growth factor receptor 3 in the recurrence of non-muscle-invasive urothelial carcinoma of the bladder. Korean J Urol. 2010; 51:94–100. 10.4111/kju.2010.51.2.94 20414420 PMC2855482

[36] Seo HK, Cho KS, Chung J, Joung JY, Park WS, Chung MK, et al Prognostic value of p53 and Ki-67 expression in intermediate-risk patients with nonmuscle-invasive bladder cancer receiving adjuvant intravesical mitomycin C therapy. Urology. 2010; 76:512 e1-7. 10.1016/j.urology.2010.04.040 20579709

[37] Behnsawy HM, Miyake H, Abdalla MA, Sayed MA, Ahmed Ael F, Fujisawa M. Expression of cell cycle-associated proteins in non-muscle-invasive bladder cancer: correlation with intravesical recurrence following transurethral resection. Urol Oncol. 2011; 29:495–501. 10.1016/j.urolonc.2009.08.002 19914103

[38] Wosnitzer MS, Domingo-Domenech J, Castillo-Martin M, Ritch C, Mansukhani M, Petrylack DP, et al Predictive value of microtubule associated proteins tau and stathmin in patients with nonmuscle invasive bladder cancer receiving adjuvant intravesical taxane therapy. J Urol. 2011; 186:2094–100. 10.1016/j.juro.2011.06.051 21944130

[39] Youssef RF, Shariat SF, Kapur P, Kabbani W, Ghoneim T, King E, et al Expression of cell cycle-related molecular markers in patients treated with radical cystectomy for squamous cell carcinoma of the bladder. Hum Pathol. 2011; 42:347–55. 10.1016/j.humpath.2010.07.012 21111452

[40] Bi J, Chen X, Zhang Y, Li B, Sun J, Shen H, et al Fascin is a predictor for invasiveness and recurrence of urothelial carcinoma of bladder. Urol Oncol. 2012; 30:688–94. 10.1016/j.urolonc.2010.08.001 20888270

[41] Shan GY, Zhang Z, Chen QG, Yu XY, Liu GB, Kong CZ. Overexpression of RIN1 associates with tumor grade and progression in patients of bladder urothelial carcinoma. Tumour Biol. 2012; 33:847–55. 10.1007/s13277-011-0311-1 22249975

[42] Oderda M, Ricceri F, Pisano F, Fiorito C, Gurioli A, Casetta G, et al Prognostic factors including Ki-67 and p53 in Bacillus Calmette-Guerin-treated non-muscle-invasive bladder cancer: a prospective study. Urol Int. 2013; 90:184–90. 10.1159/000343431 23328160

[43] Otto W, Denzinger S, Fritsche HM, Burger M, Rossler W, Bertz S, et al Introduction and first clinical application of a simplified immunohistochemical validation system confirms prognostic impact of KI-67 and CK20 for stage T1 urothelial bladder carcinoma: single-center analysis of eight biomarkers in a series of three hundred six patients. Clin Genitourin Cancer. 2013; 11:537–44. 10.1016/j.clgc.2013.05.001 23850551

[44] Park J, Song C, Shin E, Hong JH, Kim CS, Ahn H. Do molecular biomarkers have prognostic value in primary T1G3 bladder cancer treated with bacillus Calmette-Guerin intravesical therapy? Urol Oncol. 2013; 31:849–56. 10.1016/j.urolonc.2011.06.004 21782482

[45] Ruan J, Wei B, Xu Z, Yang S, Zhou Y, Yu M, et al Predictive value of Sox2 expression in transurethral resection specimens in patients with T1 bladder cancer. Med Oncol. 2013; 30:445 10.1007/s12032-012-0445-z 23307254

[46] Bertz S, Otto W, Denzinger S, Wieland WF, Burger M, Stohr R, et al Combination of CK20 and Ki-67 immunostaining analysis predicts recurrence, progression, and cancer-specific survival in pT1 urothelial bladder cancer. Eur Urol. 2014; 65:218–26. 10.1016/j.eururo.2012.05.033 22633802

[47] Wang LC, Xylinas E, Kent MT, Kluth LA, Rink M, Jamzadeh A, et al Combining smoking information and molecular markers improves prognostication in patients with urothelial carcinoma of the bladder. Urol Oncol. 2014; 32:433–40. 10.1016/j.urolonc.2013.10.015 24433754

[48] Ding W, Gou Y, Sun C, Xia G, Wang H, Chen Z, et al Ki-67 is an independent indicator in non-muscle invasive bladder cancer (NMIBC); combination of EORTC risk scores and Ki-67 expression could improve the risk stratification of NMIBC. Urol Oncol. 2014; 32:42 e13-9. 10.1016/j.urolonc.2013.05.004 24360660

[49] Poyet C, Buser L, Roudnicky F, Detmar M, Hermanns T, Mannhard D, et al Connexin 43 expression predicts poor progression-free survival in patients with non-muscle invasive urothelial bladder cancer. J Clin Pathol. 2015; 68:819–24. 10.1136/jclinpath-2015-202898 26251520 PMC4602233

[50] Wang L, Zhou M, Feng C, Gao P, Ding G, Zhou Z, et al Prognostic value of Ki67 and p63 expressions in bladder cancer patients who underwent radical cystectomy. Int Urol Nephrol. 2016; 48:495–501. 10.1007/s11255-015-1197-4 26759323

[51] Schepeler T, Lamy P, Hvidberg V, Laurberg JR, Fristrup N, Reinert T, et al A high resolution genomic portrait of bladder cancer: correlation between genomic aberrations and the DNA damage response. Oncogene. 2013; 32:3577–86. 10.1038/onc.2012.381 22926521

[52] Pichu S, Krishnamoorthy S, Shishkov A, Zhang B, McCue P, Ponnappa BC. Knockdown of Ki-67 by dicer-substrate small interfering RNA sensitizes bladder cancer cells to curcumin-induced tumor inhibition. PLoS One. 2012; 7:e48567 10.1371/journal.pone.0048567 23152782 PMC3495973

[53] Wang L, Feng C, Ding G, Ding Q, Zhou Z, Jiang H, et al Ki67 and TP53 expressions predict recurrence of non-muscle-invasive bladder cancer. Tumour Biol. 2014; 35:2989–95. 10.1007/s13277-013-1384-9 24241960

[54] Bryan RT, Zeegers MP, James ND, Wallace DM, Cheng KK. Biomarkers in bladder cancer. BJU Int. 2010; 105:608–13. 10.1111/j.1464-410X.2009.08880.x 19793380

[55] Lan YJ, Chen H, Chen JQ, Lei QH, Zheng M, Shao ZR. Immunolocalization of vimentin, keratin 17, Ki-67, involucrin, beta-catenin and E-cadherin in cutaneous squamous cell carcinoma. Pathol Oncol Res. 2014; 20:263–6. 10.1007/s12253-013-9690-5 23999979

[56] da Silva SD, Morand GB, Alobaid FA, Hier MP, Mlynarek AM, Alaoui-Jamali MA, et al Epithelial-mesenchymal transition (EMT) markers have prognostic impact in multiple primary oral squamous cell carcinoma. Clin Exp Metastasis. 2015; 32:55–63. 10.1007/s10585-014-9690-1 25433796

[57] Islam SS, Mokhtari RB, Noman AS, Uddin M, Rahman MZ, Azadi MA, et al Sonic hedgehog (Shh) signaling promotes tumorigenicity and stemness via activation of epithelial-to-mesenchymal transition (EMT) in bladder cancer. Mol Carcinog. 2016; 55:537–51. 10.1002/mc.22300 25728352

[58] Yu JQ, Zhou Q, Zheng YF, Bao Y. Expression of Vimentin and Ki-67 Proteins in Cervical Squamous Cell Carcinoma and their Relationships with Clinicopathological Features. Asian Pac J Cancer Prev. 2015; 16:4271–5. 26028085 10.7314/apjcp.2015.16.10.4271

