# Supplementary figures and images for: Clinicopathological and Prognostic Value of Ki-67 Expression in Bladder Cancer: A Systematic Review and Meta-Analysis
Source: PLoS One. 2016 Jul 13;11(7):e0158891. doi: 10.1371/journal.pone.0158891 (PMC4943634; doi:10.1371/journal.pone.0158891)

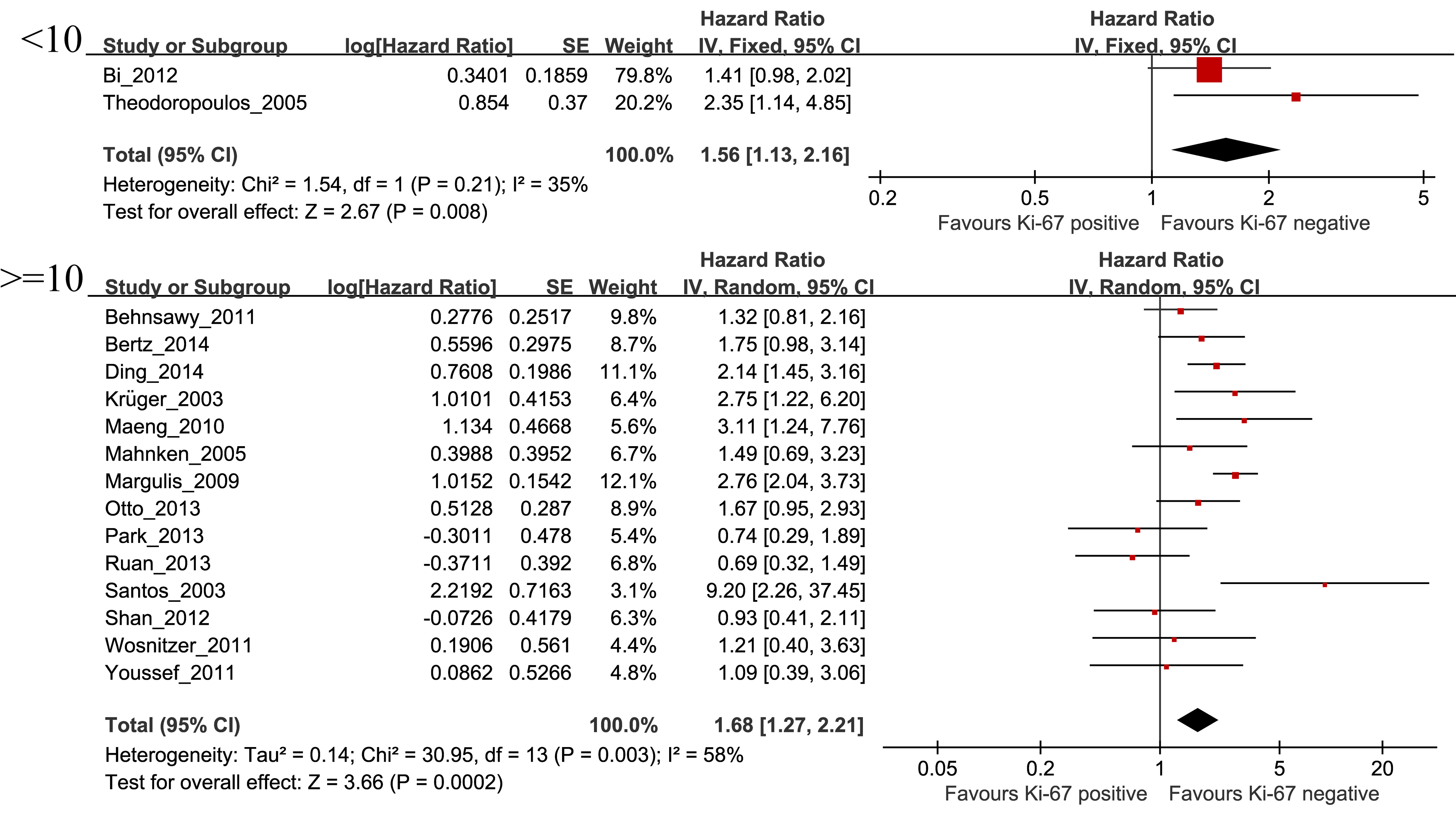

Supplement: S1 Fig — HR of Ki-67 expression associated with RFS in all BC patients subgroup. Abbreviations: HR, hazard ratio; RFS, recurrence-free survival; BC, bladder cancer. (TIF) [file pone.0158891.s001.tif]

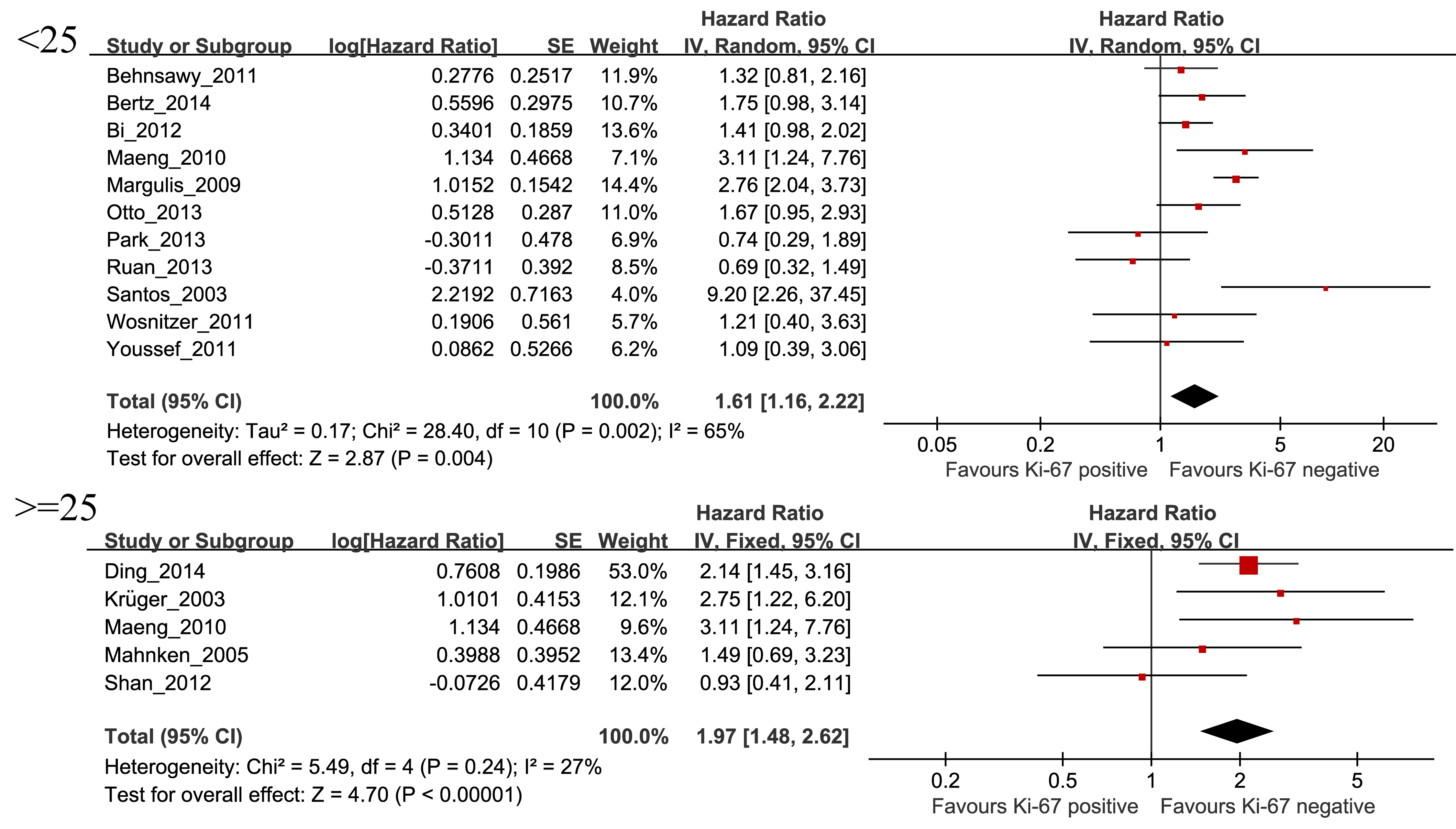

Supplement: S2 Fig — . HR of Ki-67 expression associated with RFS in all BC patients subgroup. Abbreviations: HR, hazard ratio; RFS, recurrence-free survival; BC, bladder cancer. (TIF) [file pone.0158891.s002.tif]

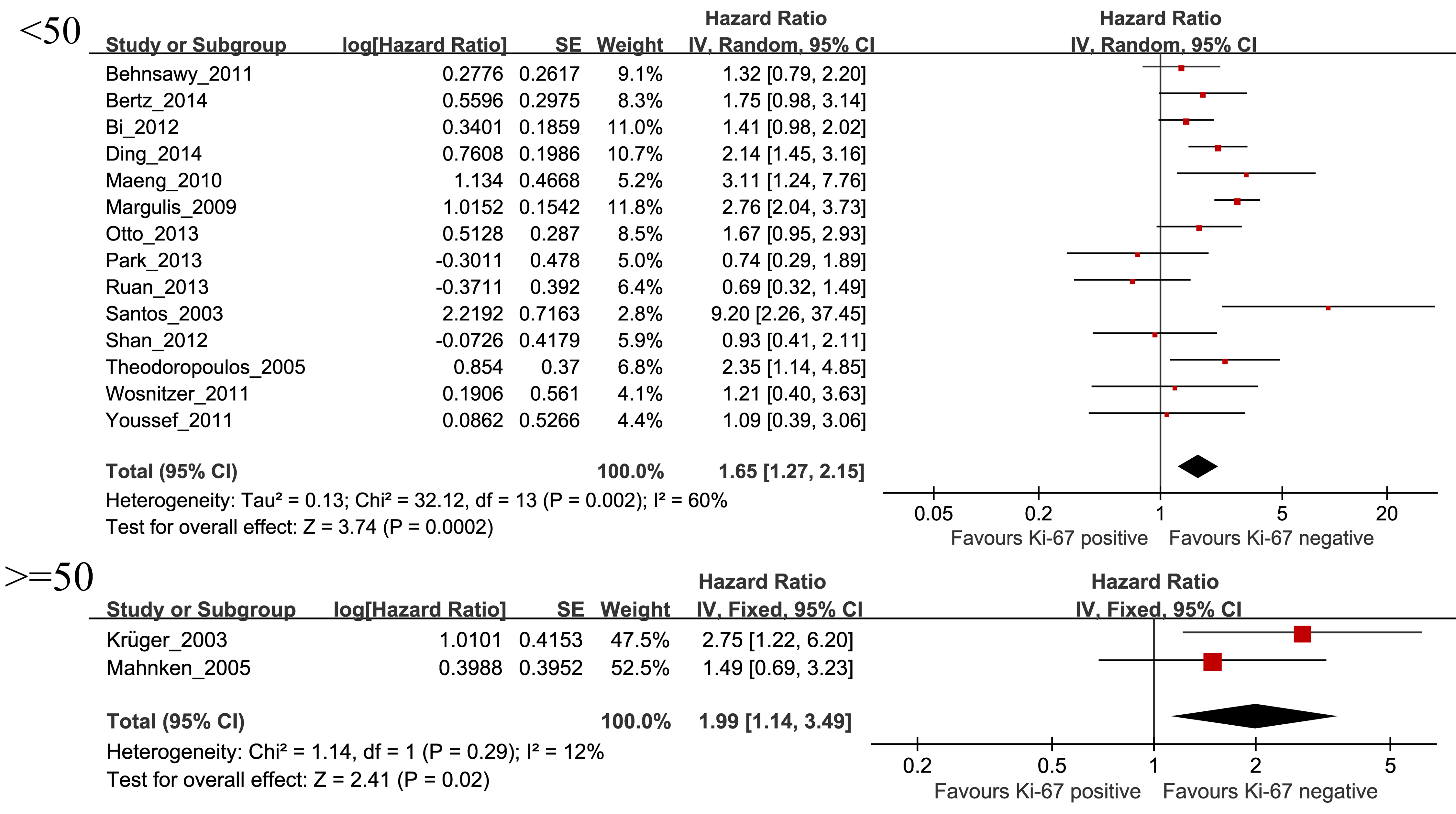

Supplement: S3 Fig — . HR of Ki-67 expression associated with RFS in all BC patients subgroup. Abbreviations: HR, hazard ratio; RFS, recurrence-free survival; BC, bladder cancer. (TIF) [file pone.0158891.s003.tif]

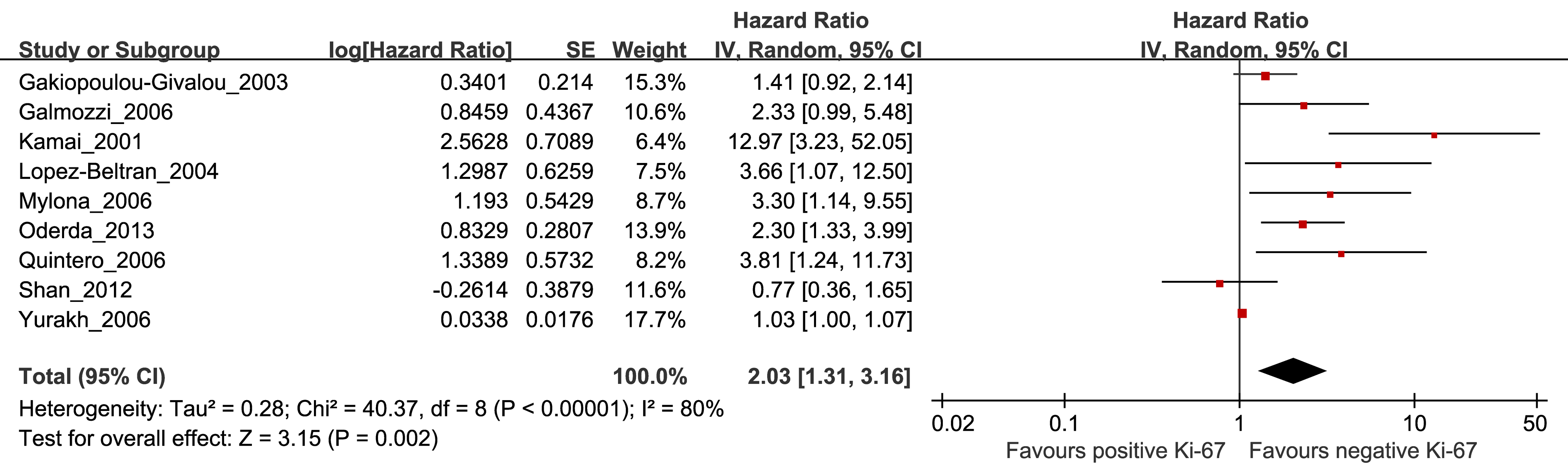

Supplement: S4 Fig — Abbreviations: HR, hazard ratio; OS, overall survival; BC, bladder cancer. (TIF) [file pone.0158891.s004.tif]

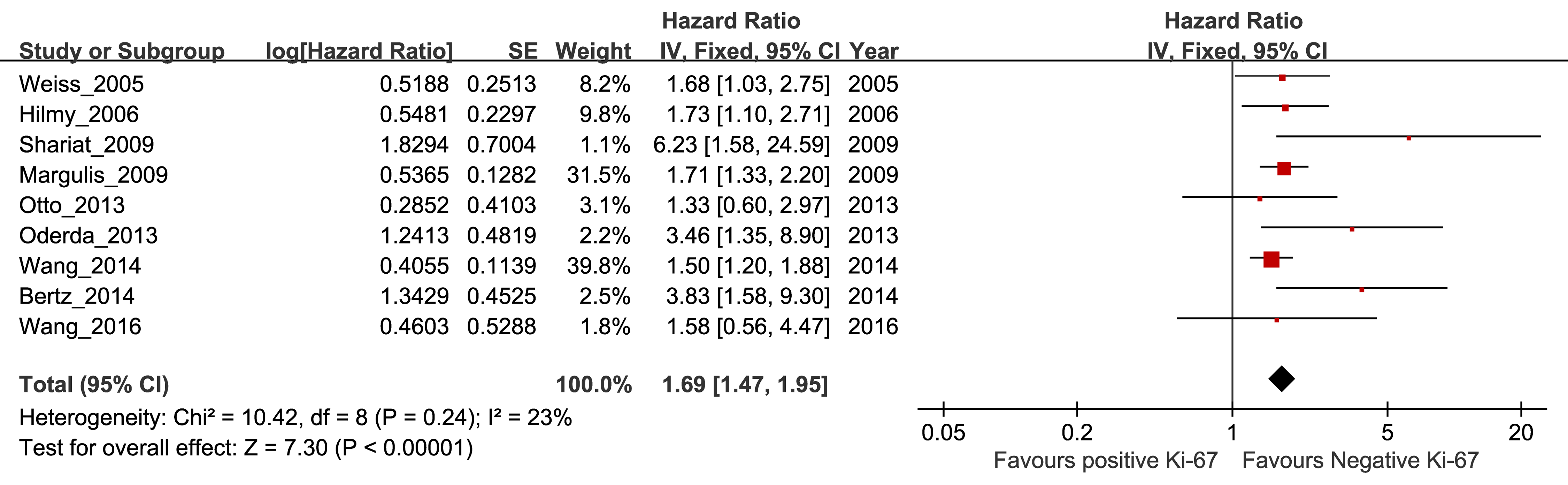

Supplement: S5 Fig — Abbreviations: HR, hazard ratio; CSS, cancer-specific survival; BC, bladder cancer. (TIF) [file pone.0158891.s005.tif]
