# Supplementary material for: Clinicopathological and Prognostic Value of Ki-67 Expression in Bladder Cancer: A Systematic Review and Meta-Analysis
Source: PLoS One. 2016 Jul 13;11(7):e0158891. doi: 10.1371/journal.pone.0158891 (PMC4943634; doi:10.1371/journal.pone.0158891)
Supplement: S1 Table — (DOCX) [file pone.0158891.s006.docx]

**Supplement Table 1. Summary of the characteristics of enrolled studies**

| **Study(year)** | **Country** | **Patients (n)** | **Age (year)** | **Gender (M/F)** | **Antibody and dilution** | **Cut-off (%)** | **Follow up (month)** | **Endpoint** | **RFS/PFS/OS/CSS HR (95%CI)** | **NOS** |
| --- | --- | --- | --- | --- | --- | --- | --- | --- | --- | --- |
| Kamai_2001 | Japan | 145 | Mean 76.1 | 101/44 | MIB-1; NA | 30 | Median 50 | OS | O: 12.87(3.23-52.06) | 6 |
| Krüger_2003 | Germany | 54 | Median 68 | 46/8 | MIB-1; 1:20 | 50 | Median 43 | RFS, PFS | R: 2.75(1.22-6.18)  P: 1.47(0.48-4.49) | 6 |
| Santos_2003 | Portugal | 56 | Median 70 | 40/16 | MIB-1; 1:50 | 20 | Median 76.7 | RFS | R: 9.20( 2.62-32.60) | 7 |
| Gakiopoulou-Givalou_2003 | Greece | 114 | Mean 69.29 | 97/17 | Anti-ki67; 1:200 | 10 | Median 46 | OS | O: 1.41(0.92-2.14) | 6 |
| Lopez-Beltran_2004 | Spain | 159 | Mean 61 | 141/18 | MIB-1; NA | 13 | Mean 74.8 | OS | O: 3.66(1.07-12.50) | 7 |
| Mahnken_2005 | Germany | 69 | Median 68 | 57/12 | MIB-1; 1:20 | 50 | NA | RFS, PFS | R: 1.49(0.69-3.24)  P: 1.13(0.50-2.59) | 6 |
| Theodoropoulos_2005 | Greece | 140 | Mean 69 | 107/33 | MIB-1; 1:10 | 8.6 | Median 41 | RFS | R: 2.35(1.14-4.85) | 8 |
| Weiss_2005 | Germany | 134 | Median 66 | 110/24 | MIB-1; 1:50 | 14.2 | Median 49.5 | CSS | C: 1.68(1.03-2.74) | 8 |
| Mylona_2006 | Greece | 123 | Mean 68.99 | 107/16 | MIB-1; 1:50 | 10 | Median 82.24 | OS | O: 3.30(1.14-9.57) | 7 |
| Galmozzi_2006 | Italy | 82 | NA | 73/9 | Anti-ki67; NA | 55 | Median 21 | OS | O: 2.33(0.99-5.43) | 8 |
| Hilmy_2006 | UK | 103 | NA | 70/33 | MIB-1; NA | NA | Median 60 | CSS | C: 1.73(1.11-2.72) | 6 |
| Quintero_2006 | Spain | 164 | Mean 61 | 143/21 | MIB-1; NA | 13 | Mean 75 | PFS, OS | P: 3.38(1.25-9.14)  O: 3.81(1.24-11.73) | 8 |
| Yurakh_2006 | Spain | 84 | NA | 72/12 | MIB-1; 1:50 | 50 | Median 36.4 | OS | O: 1.03(1.00–1.07) | 6 |
| Shariat_2009 | USA | 80 | Median 64.6 | 61/19 | MIB-1; 1:500 | 20 | Median 61.6 | CSS | C: 6.23(1.58-24.48) | 6 |
| Margulis_2009 | USA | 713 | Mean 67.5 | 570/143 | Anti-ki67; NA | 20 | Median 57.6 | RFS, CSS | R: 2.76(2.04-3.73)  C: 1.71(1.33-2.21) | 8 |
| Maeng_2010 | Korea | 55 | Mean 67 | 40/15 | Anti-ki67; 1:80 | 25 | Median 26.2 | RFS | R: 3.11(1.24-7.76) | 8 |
| Seo_2010 | Korea | 129 | Median 64.2 | 104/25 | MIB-1; 1:50 | 20 | Mean 48.6 | PFS | P: 3.40(1.04-11.05) | 7 |
| Behnsawy_2011 | Japan | 161 | NA | 137/24 | Anti-ki67; 1:200 | 10 | Median 47 | RFS | R: 1.32(0.46-1.24) | 7 |
| Wosnitzer_2011 | USA | 32 | Median 70.3 | 25/7 | MIB-1; NA | 10 | Median 22 | RFS | R: 1.21(0.40-3.67) | 8 |
| Youssef_2011 | USA | 152 | Median 51 | 99/53 | MIB-1; 1:500 | 20 | Median 63.2 | RFS | R: 1.09(0.39-3.08) | 8 |
| Bi_2012 | China | 111 | Median 66 | 93/18 | Anti-ki67; 1:400 | 5 | Mean 13 | RFS | R: 1.41(0.98-2.02) | 7 |
| Shan_2012 | China | 96 | Mean 55 | 69/27 | MIB-1; NA | 41 | Median 49 | RFS,PFS, OS | R: 0.93(0.41-2.11)  P: 0.57(0.22-1.49)  O: 0.77(0.36-1.61) | 7 |
| Oderda_2013 | Italy | 192 | Mean 73.2 | 166/26 | MIB-1; 1:10 | 20 | Median 100 | OS, CSS | O: 2.30(1.33-3.99)  C: 3.46(1.35-8.90) | 7 |
| Otto_2013 | Germany | 306 | Median 72 | 238/68 | MIB-1; 1:200 | 10 | Median 39 | RFS, PFS, CSS | R: 1.67(0.95-2.93)  P: 2.11(1.02-4.37)  C: 1.33(0.60-2.96) | 6 |
| Park_2013 | Korea | 61 | Median 66 | 53/8 | Anti-Ki-67; 1:200 | 10.4 | Median 60 | RFS, PFS | R: 0.74(0.29-1.87)  P: 0.42(0.08-2.12) | 7 |
| Ruan_2013 | China | 126 | Mean 64.52 | 103/23 | Anti-Ki-67; 1:50 | 10 | NA | RFS | R: 0.69(0.32-1.49) | 7 |
| Bertz_2014 | Germany | 309 | Median 71.7 | 237/72 | MIB-1; 1:50 | 15 | Median 49 | RFS, PFS, CSS | R: 1.75(0.98-3.15)  P: 2.80(1.45-5.43)  C: 3.83(1.59-9.26) | 6 |
| Wang_2014 | USA | 588 | Median 65 | 494/94 | MIB-1; 1:500 | 20 | NA | CSS | C: 1.5(1.2-2.1) | 6 |
| Ding_2014 | China | 332 | Median 67 | 273/59 | Anti-Ki-67; 1:100 | 25 | Median 47 | RFS,PFS | R: 2.14(1.45-3.16)  P: 2.97(1.42-6.22) | 8 |
| Poyet_2015 | Switzerland | 174 | Median 69.5 | 131/43 | MIB-1; 1:50 | 10 | Median 110.6 | PFS | P: 1.76(0.63-4.94) | 8 |
| Wang_2016 | China | 103 | Mean 65.85 | 78/25 | Anti-Ki-67; 1:100 | 40 | Mean 22 | CSS | C: 1.58(0.56-4.47) | 6 |

CSS: cancer-specific survival; F: female; HR: hazard ratio; M: male; NA: not available; NOS: Newcastle-Ottawa Scale; OS: overall survival; PFS: progression-free survival; RFS: recurrence-free survival.
