# Supplementary material for: Clinicopathological and Prognostic Value of Ki-67 Expression in Bladder Cancer: A Systematic Review and Meta-Analysis
Source: PLoS One. 2016 Jul 13;11(7):e0158891. doi: 10.1371/journal.pone.0158891 (PMC4943634; doi:10.1371/journal.pone.0158891)
Supplement: S2 Table — (DOCX) [file pone.0158891.s007.docx]

**Supplement Table 2. HR values of RFS of BC subgroups depended on cutoff value**

| **Cutoff Value (%)** | **Studies（n）** | **HR** | **95%CI** | **P value** | **Model** | **Heterogeneity** |
| --- | --- | --- | --- | --- | --- | --- |
|  |  |  |  |  |  | **Chi^2^, I^2^, P value** |
| **<10** | 2 | 1.56 | 1.13-2.16 | 0.008 | Fixed | 1.54, 35%, 0.21 |
| **≥10** | 14 | 1.68 | 1.27-2.21 | 0.0002 | Random | 30.95, 58%, 0.003 |
| **<25** | 11 | 1.61 | 1.16-2.22 | 0.004 | Random | 28.40, 65%, 0.002 |
| **≥25** | 5 | 1.97 | 1.48-2.62 | 0.000 | Fixed | 5.49, 27%, 0.24 |
| **<50** | 14 | 1.65 | 1.27-2.15 | 0.0002 | Random | 32.12, 60%, 0.002 |
| **≥50** | 2 | 1.99 | 1.14-3.49 | 0.02 | Fixed | 1.14, 12%, 0.29 |

HR: hazard ratio; I^2^: I-squared; Fixed: Fixed, Inverse Variance model; Random: Random, I-V heterogeneity model.
